# Disodium 4,4′-oxydibenzoate

**DOI:** 10.1107/S1600536808024331

**Published:** 2008-08-06

**Authors:** Yuan Yang, Qi Li, Seik Weng Ng

**Affiliations:** a School of Physics and Chemistry, Guizhou Normal University, Guiyang, Guizhou 550001, People’s Republic of China; b College of Chemistry, Beijing Normal University, Beijing 100875, People’s Republic of China; c Department of Chemistry, University of Malaya, 50603 Kuala Lumpur, Malaysia

## Abstract

The crystal structure of the title compound, 2Na^+^·C_14_H_8_O_5_^2−^, consists of alternating layers of sodium cations and 4,4′-oxydibenzoate anions; the layers are perpendicular to the *a* axis, with the distance between the layers of cations (or anions) being half this axial length. The Na atoms are disordered over three sites [occupancies 0.775 (4), 0.781 (6) 0.444 (6)].

## Related literature

For the crystal structure of 4,4′-oxybis(benzoic acid), see: Dey & Desiraju (2005 ▶); Potts *et al.* (2007 ▶).
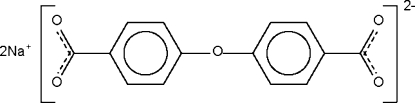


## Experimental

### 

#### Crystal data

2Na^+^·C_14_H_8_O_5_^2−^
*M* *_r_* = 302.18
Monoclinic, 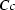

*a* = 29.1091 (4) Å
*b* = 5.7801 (1) Å
*c* = 7.6429 (1) Å
β = 92.4420 (1)°
*V* = 1284.78 (3) Å^3^
*Z* = 4
Mo *K*α radiation
μ = 0.17 mm^−1^
*T* = 295 (2) K
0.5 × 0.4 × 0.2 mm

#### Data collection

Bruker SMART APEXII diffractometer
Absorption correction: multi-scan (*SADABS*; Sheldrick, 1996 ▶) *T*_min_ = 0.888, *T*_max_ = 1.000 (expected range = 0.857–0.966)
5002 measured reflections
1471 independent reflections
1464 reflections with *I* > 2σ(*I*)
*R*_int_ = 0.015

#### Refinement

*R*[*F* ^2^ > 2σ(*F* ^2^)] = 0.042
*wR*(*F* ^2^) = 0.135
*S* = 1.27
1471 reflections
203 parameters
3 restraints
H-atom parameters constrained
Δρ_max_ = 0.79 e Å^−3^
Δρ_min_ = −0.27 e Å^−3^

### 

Data collection: *APEX2* (Bruker, 2007 ▶); cell refinement: *SAINT* (Bruker, 2007 ▶); data reduction: *SAINT*; program(s) used to solve structure: *SHELXS97* (Sheldrick, 2008 ▶); program(s) used to refine structure: *SHELXL97* (Sheldrick, 2008 ▶); molecular graphics: *X-SEED* (Barbour, 2001 ▶); software used to prepare material for publication: *publCIF* (Westrip, 2008 ▶).

## Supplementary Material

Crystal structure: contains datablocks global, I. DOI: 10.1107/S1600536808024331/bt2752sup1.cif

Structure factors: contains datablocks I. DOI: 10.1107/S1600536808024331/bt2752Isup2.hkl

Additional supplementary materials:  crystallographic information; 3D view; checkCIF report

## Figures and Tables

**Fig. 1.**
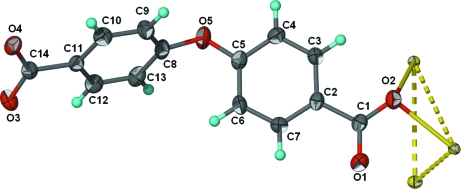
Thermal ellipsoid plot (Barbour, 2001) of the asymmetric unit of the title compound; displac ement ellipsoids are set at the 70% probability level. Hydrogeatoms are drawn as spheres of arbitrary radius. The two sodium atoms are disordered over three positions. Dashed lines denote the distances between them.

## supplementary crystallographic information

### Comment

The crystal structure of disodium 4,4'-oxydibenzoate (Scheme I, Fig. 1) consists of alternating bands of sodium cations and 4,4'-oxydibenzoate anions. The two symmetry-independent sodium atoms over three positions. The lowest occupancy sodium atom is only weakly linked to two oxygen atoms, and probably "rattles" about in the crystal structure.

### Experimental

Betaine (0.047 g, 0.4 mmol), 4,4'-oxybis(benzoic acid) (0.103 g, 0.4 mmol) and guanidine hydrochloride (0.076 g, 0.8 mmol) were mixed in a molar ratio 1:1:2. The mixture was dissolved in mixture of ethanol (4 ml), 1 *M* sodium hydroxide (0.5 ml) and water (0.5 ml). Colorless crystals were obtained after about 10 days.

### Refinement

The Na1, Na2 and Na3 atoms were refined such that the total occupancy is two.

Carbon  bound H-atoms were placed in calculated positions (C—H 0.93 Å) and were included in the refinement in the riding model approximation, with *U*_iso_(H) 1.2*U*_eq_(C).

Friedel pairs were merged as there are no anomalous scatterers.

### Crystal data

**Table:**

2Na^+^·C_14_H_8_O_5_^2–^	*F*_000_ = 616
*M**_r_* = 302.18	*D*_x_ = 1.562 Mg m^−^^3^
Monoclinic, *C**c*	Mo *K*α radiation λ = 0.71073 Å
*a* = 29.1091 (4) Å	Cell parameters from 4314 reflections
*b* = 5.7801 (1) Å	θ = 4.0–27.5º
*c* = 7.6429 (1) Å	µ = 0.18 mm^−^^1^
β = 92.4420 (1)º	*T* = 295 (2) K
*V* = 1284.78 (3) Å^3^	Block, colorless
*Z* = 4	0.5 × 0.4 × 0.2 mm

### Data collection

**Table:**

Bruker SMART APEXII diffractometer	1471 independent reflections
Radiation source: fine-focus sealed tube	1464 reflections with *I* > 2σ(*I*)
Monochromator: graphite	*R*_int_ = 0.015
*T* = 295(2) K	θ_max_ = 27.5º
φ and ω scans	θ_min_ = 4.1º
Absorption correction: multi-scan(SADABS; Sheldrick, 1996)	*h* = −37→37
*T*_min_ = 0.888, *T*_max_ = 1.000	*k* = −7→7
5002 measured reflections	*l* = −9→9

### Refinement

**Table:**

Refinement on *F*^2^	Secondary atom site location: difference Fourier map
Least-squares matrix: full	Hydrogen site location: inferred from neighbouring sites
*R*[*F*^2^ > 2σ(*F*^2^)] = 0.042	H-atom parameters constrained
*wR*(*F*^2^) = 0.135	*w* = 1/[σ^2^(*F*_o_^2^) + (0.0883*P*)^2^ + 0.9438*P*] where *P* = (*F*_o_^2^ + 2*F*_c_^2^)/3
*S* = 1.27	(Δ/σ)_max_ = 0.001
1471 reflections	Δρ_max_ = 0.79 e Å^−^^3^
203 parameters	Δρ_min_ = −0.27 e Å^−^^3^
3 restraints	Extinction correction: SHELXL97 (Sheldrick, 2008), Fc^*^=kFc[1+0.001xFc^2^λ^3^/sin(2θ)]^-1/4^
Primary atom site location: structure-invariant direct methods	Extinction coefficient: 0.024 (4)

### Special details

**Table:**

Experimental. A somewhat large crystal was used in the measurements, but this does not seem to have an adverse efffect on the quality of the diffraction data.

### Fractional atomic coordinates and isotropic or equivalent isotropic displacement parameters (Å^2^)

**Table:**

	*x*	*y*	*z*	*U*_iso_*/*U*_eq_	Occ. (<1)
Na1	0.50001 (5)	0.1573 (3)	0.50001 (18)	0.0119 (4)	0.775 (4)
Na2	0.44106 (5)	0.3880 (3)	0.84063 (18)	0.0113 (5)	0.781 (6)
Na3	0.47490 (10)	0.7168 (5)	0.7153 (4)	0.0147 (9)	0.444 (6)
O1	0.53701 (10)	0.4833 (5)	0.9932 (4)	0.0273 (6)	
O2	0.51425 (9)	0.1864 (5)	0.8264 (4)	0.0243 (6)	
O3	0.91491 (10)	0.5648 (5)	0.9768 (4)	0.0279 (6)	
O4	0.92132 (9)	0.1917 (5)	1.0487 (4)	0.0249 (6)	
O5	0.72485 (10)	0.1735 (6)	0.6575 (4)	0.0338 (7)	
C1	0.54493 (12)	0.3222 (6)	0.8891 (5)	0.0199 (7)	
C2	0.59376 (12)	0.2835 (6)	0.8364 (4)	0.0199 (7)	
C3	0.60419 (13)	0.0883 (6)	0.7386 (5)	0.0234 (7)	
H3	0.5812	−0.0182	0.7088	0.028*	
C4	0.64896 (13)	0.0513 (7)	0.6849 (5)	0.0250 (7)	
H4	0.6559	−0.0793	0.6201	0.030*	
C5	0.68284 (12)	0.2121 (7)	0.7296 (5)	0.0230 (7)	
C6	0.67343 (13)	0.4048 (7)	0.8307 (5)	0.0251 (8)	
H6	0.6966	0.5089	0.8634	0.030*	
C7	0.62823 (12)	0.4391 (7)	0.8825 (5)	0.0228 (7)	
H7	0.6214	0.5685	0.9488	0.027*	
C8	0.76546 (12)	0.2304 (8)	0.7501 (5)	0.0261 (8)	
C9	0.78630 (13)	0.0650 (7)	0.8574 (5)	0.0266 (8)	
H9	0.7713	−0.0733	0.8792	0.032*	
C10	0.82982 (14)	0.1068 (7)	0.9322 (5)	0.0248 (7)	
H10	0.8442	−0.0052	1.0026	0.030*	
C11	0.85196 (11)	0.3161 (6)	0.9022 (4)	0.0194 (7)	
C12	0.82984 (13)	0.4838 (7)	0.7983 (5)	0.0243 (7)	
H12	0.8440	0.6256	0.7813	0.029*	
C13	0.78638 (13)	0.4407 (7)	0.7192 (5)	0.0264 (7)	
H13	0.7719	0.5509	0.6475	0.032*	
C14	0.89941 (12)	0.3616 (6)	0.9821 (5)	0.0200 (7)	

### Atomic displacement parameters (Å^2^)

**Table:**

	*U*^11^	*U*^22^	*U*^33^	*U*^12^	*U*^13^	*U*^23^
Na1	0.0114 (7)	0.0098 (7)	0.0144 (7)	0.0005 (5)	−0.0009 (5)	−0.0005 (5)
Na2	0.0119 (8)	0.0094 (7)	0.0127 (8)	−0.0011 (5)	0.0003 (5)	0.0001 (5)
Na3	0.0169 (14)	0.0144 (15)	0.0127 (14)	0.0013 (10)	0.0008 (10)	−0.0004 (10)
O1	0.0230 (11)	0.0294 (14)	0.0299 (15)	0.0020 (11)	0.0043 (10)	−0.0037 (11)
O2	0.0183 (11)	0.0254 (13)	0.0293 (13)	−0.0020 (9)	0.0007 (10)	0.0005 (10)
O3	0.0262 (13)	0.0292 (15)	0.0282 (14)	−0.0081 (11)	−0.0015 (11)	0.0009 (11)
O4	0.0209 (12)	0.0281 (13)	0.0254 (13)	0.0042 (10)	−0.0017 (10)	0.0008 (10)
O5	0.0171 (12)	0.056 (2)	0.0288 (14)	−0.0011 (12)	0.0005 (10)	−0.0141 (13)
C1	0.0181 (15)	0.0215 (15)	0.0200 (15)	0.0019 (12)	0.0015 (12)	0.0039 (12)
C2	0.0165 (15)	0.0228 (16)	0.0204 (15)	0.0011 (12)	−0.0006 (12)	0.0030 (12)
C3	0.0187 (16)	0.0241 (17)	0.0274 (18)	−0.0015 (13)	0.0007 (13)	−0.0029 (13)
C4	0.0223 (17)	0.0260 (17)	0.0267 (17)	0.0035 (15)	0.0008 (13)	−0.0051 (14)
C5	0.0179 (16)	0.0299 (17)	0.0211 (15)	0.0015 (13)	0.0003 (12)	−0.0001 (13)
C6	0.0174 (16)	0.0290 (19)	0.0287 (17)	−0.0044 (13)	−0.0014 (13)	−0.0039 (15)
C7	0.0226 (17)	0.0217 (16)	0.0243 (17)	−0.0001 (13)	0.0016 (13)	−0.0034 (12)
C8	0.0159 (16)	0.039 (2)	0.0232 (17)	−0.0016 (14)	0.0031 (13)	−0.0074 (15)
C9	0.0227 (17)	0.0278 (18)	0.0294 (18)	−0.0069 (14)	0.0037 (14)	−0.0022 (14)
C10	0.0260 (18)	0.0237 (16)	0.0247 (17)	−0.0007 (14)	0.0024 (13)	0.0025 (14)
C11	0.0162 (15)	0.0232 (16)	0.0187 (15)	0.0003 (11)	0.0008 (11)	−0.0018 (12)
C12	0.0237 (17)	0.0238 (17)	0.0255 (18)	−0.0004 (13)	0.0028 (13)	0.0033 (13)
C13	0.0210 (16)	0.033 (2)	0.0252 (16)	0.0056 (15)	−0.0003 (12)	0.0026 (15)
C14	0.0188 (16)	0.0252 (16)	0.0162 (14)	−0.0013 (12)	0.0023 (12)	−0.0001 (12)

### Geometric parameters (Å, °)

**Table:**

Na1—O1^i^	2.342 (3)	O5—C8	1.391 (5)
Na1—O2^ii^	2.434 (3)	C1—C2	1.511 (5)
Na1—O4^iii^	2.494 (3)	C2—C7	1.382 (5)
Na1—O2	2.517 (3)	C2—C3	1.394 (5)
Na1—O3^iii^	2.789 (3)	C3—C4	1.399 (5)
Na2—O3^iv^	2.285 (3)	C3—H3	0.9300
Na2—O4^iii^	2.326 (3)	C4—C5	1.388 (5)
Na2—O2	2.435 (3)	C4—H4	0.9300
Na2—O4^v^	2.453 (3)	C5—C6	1.389 (6)
O1—C1	1.252 (5)	C6—C7	1.404 (5)
O1—Na1^vi^	2.342 (3)	C6—H6	0.9300
O1—Na3^vi^	2.784 (4)	C7—H7	0.9300
O2—C1	1.268 (4)	C8—C9	1.382 (6)
O2—Na1^vii^	2.434 (3)	C8—C13	1.384 (6)
O3—C14	1.260 (5)	C9—C10	1.389 (5)
O3—Na2^viii^	2.285 (3)	C9—H9	0.9300
O3—Na3^ix^	2.774 (4)	C10—C11	1.394 (5)
O3—Na1^x^	2.789 (3)	C10—H10	0.9300
O4—C14	1.266 (5)	C11—C12	1.393 (5)
O4—Na2^x^	2.326 (3)	C11—C14	1.509 (5)
O4—Na2^xi^	2.453 (3)	C12—C13	1.401 (5)
O4—Na1^x^	2.494 (3)	C12—H12	0.9300
O5—C5	1.381 (4)	C13—H13	0.9300
			
O1^i^—Na1—O2^ii^	128.52 (12)	O2—C1—C2	117.6 (3)
O1^i^—Na1—O4^iii^	96.84 (11)	C7—C2—C3	119.4 (3)
O2^ii^—Na1—O4^iii^	123.07 (11)	C7—C2—C1	121.1 (3)
O1^i^—Na1—O2	84.62 (11)	C3—C2—C1	119.5 (3)
O2^ii^—Na1—O2	124.75 (10)	C2—C3—C4	120.5 (3)
O4^iii^—Na1—O2	86.69 (10)	C2—C3—H3	119.7
O1^i^—Na1—O3^iii^	144.60 (11)	C4—C3—H3	119.7
O2^ii^—Na1—O3^iii^	76.17 (10)	C5—C4—C3	119.1 (3)
O4^iii^—Na1—O3^iii^	49.58 (9)	C5—C4—H4	120.4
O2—Na1—O3^iii^	101.66 (10)	C3—C4—H4	120.4
O3^iv^—Na2—O4^iii^	101.63 (12)	O5—C5—C4	115.2 (3)
O3^iv^—Na2—O2	86.45 (11)	O5—C5—C6	123.4 (3)
O4^iii^—Na2—O2	92.48 (11)	C4—C5—C6	121.3 (3)
O3^iv^—Na2—O4^v^	101.41 (12)	C5—C6—C7	118.6 (3)
O4^iii^—Na2—O4^v^	135.10 (11)	C5—C6—H6	120.7
O2—Na2—O4^v^	126.93 (11)	C7—C6—H6	120.7
O2—Na2—Na3^vi^	72.45 (9)	C2—C7—C6	121.0 (3)
C1—O1—Na1^vi^	140.4 (3)	C2—C7—H7	119.5
C1—O1—Na3^vi^	102.7 (2)	C6—C7—H7	119.5
Na1^vi^—O1—Na3^vi^	92.50 (11)	C9—C8—C13	121.6 (3)
C1—O2—Na1^vii^	115.6 (2)	C9—C8—O5	118.7 (4)
C1—O2—Na2	106.9 (2)	C13—C8—O5	119.4 (4)
Na1^vii^—O2—Na2	101.34 (11)	C8—C9—C10	119.6 (3)
C1—O2—Na1	120.0 (2)	C8—C9—H9	120.2
Na1^vii^—O2—Na1	117.45 (12)	C10—C9—H9	120.2
Na2—O2—Na1	88.21 (10)	C9—C10—C11	120.2 (4)
C14—O3—Na2^viii^	154.6 (3)	C9—C10—H10	119.9
C14—O3—Na3^ix^	128.3 (2)	C11—C10—H10	119.9
Na2^viii^—O3—Na3^ix^	73.42 (10)	C12—C11—C10	119.4 (3)
C14—O3—Na1^x^	83.5 (2)	C12—C11—C14	120.1 (3)
Na2^viii^—O3—Na1^x^	95.35 (10)	C10—C11—C14	120.4 (3)
Na3^ix^—O3—Na1^x^	68.61 (9)	C11—C12—C13	120.6 (3)
C14—O4—Na2^x^	129.7 (2)	C11—C12—H12	119.7
C14—O4—Na2^xi^	115.0 (2)	C13—C12—H12	119.7
Na2^x^—O4—Na2^xi^	115.15 (12)	C8—C13—C12	118.6 (4)
C14—O4—Na1^x^	96.8 (2)	C8—C13—H13	120.7
Na2^x^—O4—Na1^x^	91.23 (10)	C12—C13—H13	120.7
Na2^xi^—O4—Na1^x^	84.93 (10)	O3—C14—O4	124.1 (3)
C5—O5—C8	120.4 (3)	O3—C14—C11	118.2 (3)
O1—C1—O2	123.7 (3)	O4—C14—C11	117.7 (3)
O1—C1—C2	118.7 (3)		
			
O3^iv^—Na2—O2—C1	−128.5 (2)	C3—C4—C5—O5	−174.1 (4)
O4^iii^—Na2—O2—C1	130.0 (2)	C3—C4—C5—C6	1.9 (6)
O4^v^—Na2—O2—C1	−26.9 (3)	O5—C5—C6—C7	173.5 (3)
O3^iv^—Na2—O2—Na1^vii^	−7.05 (12)	C4—C5—C6—C7	−2.2 (6)
O4^iii^—Na2—O2—Na1^vii^	−108.56 (12)	C3—C2—C7—C6	0.7 (6)
O4^v^—Na2—O2—Na1^vii^	94.58 (14)	C1—C2—C7—C6	−178.9 (3)
O3^iv^—Na2—O2—Na1	110.60 (11)	C5—C6—C7—C2	0.9 (6)
O4^iii^—Na2—O2—Na1	9.09 (11)	C5—O5—C8—C9	90.4 (5)
O4^v^—Na2—O2—Na1	−147.78 (12)	C5—O5—C8—C13	−96.2 (5)
O1^i^—Na1—O2—C1	−19.8 (3)	C13—C8—C9—C10	−1.9 (6)
O2^ii^—Na1—O2—C1	114.3 (2)	O5—C8—C9—C10	171.3 (3)
O4^iii^—Na1—O2—C1	−117.0 (3)	C8—C9—C10—C11	1.3 (6)
O3^iii^—Na1—O2—C1	−164.5 (3)	C9—C10—C11—C12	0.7 (5)
C14^iii^—Na1—O2—C1	−138.8 (3)	C9—C10—C11—C14	−179.5 (3)
O1^i^—Na1—O2—Na1^vii^	−169.42 (13)	C10—C11—C12—C13	−2.2 (5)
O2^ii^—Na1—O2—Na1^vii^	−35.3 (2)	C14—C11—C12—C13	178.0 (3)
O4^iii^—Na1—O2—Na1^vii^	93.37 (13)	C9—C8—C13—C12	0.5 (6)
O3^iii^—Na1—O2—Na1^vii^	45.83 (14)	O5—C8—C13—C12	−172.7 (3)
C14^iii^—Na1—O2—Na1^vii^	71.53 (14)	C11—C12—C13—C8	1.6 (6)
O1^i^—Na1—O2—Na2	88.72 (11)	Na2^viii^—O3—C14—O4	113.0 (6)
O2^ii^—Na1—O2—Na2	−137.17 (13)	Na3^ix^—O3—C14—O4	−31.9 (5)
O4^iii^—Na1—O2—Na2	−8.48 (10)	Na1^x^—O3—C14—O4	24.2 (3)
O3^iii^—Na1—O2—Na2	−56.02 (11)	Na2^viii^—O3—C14—C11	−66.2 (7)
Na1^vi^—O1—C1—O2	−69.3 (5)	Na3^ix^—O3—C14—C11	148.8 (3)
Na3^vi^—O1—C1—O2	40.8 (4)	Na1^x^—O3—C14—C11	−155.1 (3)
Na1^vi^—O1—C1—C2	111.7 (4)	Na2^viii^—O3—C14—Na1^x^	88.8 (6)
Na3^vi^—O1—C1—C2	−138.3 (3)	Na3^ix^—O3—C14—Na1^x^	−56.1 (2)
Na1^vii^—O2—C1—O1	−88.5 (4)	Na2^x^—O4—C14—O3	70.0 (5)
Na2—O2—C1—O1	23.4 (4)	Na2^xi^—O4—C14—O3	−114.9 (4)
Na1—O2—C1—O1	121.4 (3)	Na1^x^—O4—C14—O3	−27.3 (4)
Na1^vii^—O2—C1—C2	90.6 (3)	Na2^x^—O4—C14—C11	−110.7 (3)
Na2—O2—C1—C2	−157.5 (2)	Na2^xi^—O4—C14—C11	64.4 (3)
Na1—O2—C1—C2	−59.6 (4)	Na1^x^—O4—C14—C11	152.0 (3)
O1—C1—C2—C7	−8.4 (5)	Na2^x^—O4—C14—Na1^x^	97.3 (3)
O2—C1—C2—C7	172.5 (3)	Na2^xi^—O4—C14—Na1^x^	−87.57 (17)
O1—C1—C2—C3	172.0 (4)	C12—C11—C14—O3	11.6 (5)
O2—C1—C2—C3	−7.1 (5)	C10—C11—C14—O3	−168.2 (3)
C7—C2—C3—C4	−1.0 (5)	C12—C11—C14—O4	−167.7 (3)
C1—C2—C3—C4	178.6 (3)	C10—C11—C14—O4	12.5 (5)
C2—C3—C4—C5	−0.3 (6)	C12—C11—C14—Na1^x^	−95.3 (6)
C8—O5—C5—C4	−146.1 (4)	C10—C11—C14—Na1^x^	84.9 (6)
C8—O5—C5—C6	37.9 (6)		

Symmetry codes: (i) *x*, −*y*+1, *z*−1/2; (ii) *x*, −*y*, *z*−1/2; (iii) *x*−1/2, −*y*+1/2, *z*−1/2; (iv) *x*−1/2, *y*−1/2, *z*; (v) *x*−1/2, *y*+1/2, *z*; (vi) *x*, −*y*+1, *z*+1/2; (vii) *x*, −*y*, *z*+1/2; (viii) *x*+1/2, *y*+1/2, *z*; (ix) *x*+1/2, −*y*+3/2, *z*+1/2; (x) *x*+1/2, −*y*+1/2, *z*+1/2; (xi) *x*+1/2, *y*−1/2, *z*.
